# Sustained absorption of delamanid from lipid-based formulations as a path to reduced frequency of administration

**DOI:** 10.1007/s13346-020-00851-z

**Published:** 2020-09-15

**Authors:** Gisela Ramirez, Anna C. Pham, Andrew J. Clulow, Malinda Salim, Adrian Hawley, Ben J. Boyd

**Affiliations:** 1 Drug Delivery, Disposition and Dynamics, Monash Institute of Pharmaceutical Sciences, Monash University, Parkville Campus, 381 Royal Parade, Parkville, VIC 3052 Australia; 2 SAXS/WAXS beamline, Australian Synchrotron, ANSTO, 800 Blackburn Rd, Clayton, VIC 3169 Australia; 3 ARC Centre of Excellence in Convergent Bio-Nano Science and Technology, Monash Institute of Pharmaceutical Sciences, Monash University, Parkville Campus, 381 Royal Parade, Parkville, VIC 3052 Australia

**Keywords:** Delamanid, Sustained release, Lipid formulation, Lyotropic liquid crystal, Small angle X-ray scattering, Pharmacokinetics

## Abstract

Delamanid is a poorly water-soluble drug currently being used for the treatment of tuberculosis. The high frequency of dosing leads to poor adherence for patients who live in lower economic and nomadic populations. Non-digestible self-assembling lipids as a formulation approach for poorly water-soluble drugs have previously been shown to extend the window of absorption through gastric retention. We hypothesise that this approach could lead to the reduction of dosing frequency for delamanid and thereby has potential to improve adherence. Formulations of delamanid were prepared in selachyl alcohol and phytantriol as non-digestible self-assembling lipid vehicles, and their behaviour was compared with reconstituted milk powder, as a digestible lipid-based formulation, and an aqueous suspension. The self-assembly of selachyl alcohol and phytantriol in aqueous media in the presence of delamanid was studied using small angle X-ray scattering and produced the inverse hexagonal (H_2_) and inverse bicontinuous cubic (V_2_) liquid crystal structures, respectively. The times at which maximum delamanid levels in plasma were observed (T_max_) after oral administration of the phytantriol, selachyl alcohol and reconstituted milk powder formulations of delamanid to rats were 27 ± 3, 20 ± 4 and 6.5 ± 1.0 h, respectively, compared with the aqueous suspension formulation with a T_max_ of 3.4 ± 1 h, which confirms the hypothesis of an extended duration of absorption after administration in non-digestible self-assembling lipids. The digestion products of the triglycerides in the milk formulation increased the solubilisation of delamanid in the gastrointestinal tract, leading to an increase in exposure compared with the aqueous suspension formulation but did not significantly extend T_max_. Overall, the non-digestible nanostructured lipid formulations extended the duration of absorption of delamanid well beyond that from milk or suspension formulations.

**Electronic supplementary material:**

The online version of this article (10.1007/s13346-020-00851-z) contains supplementary material, which is available to authorized users.

**Figure:**
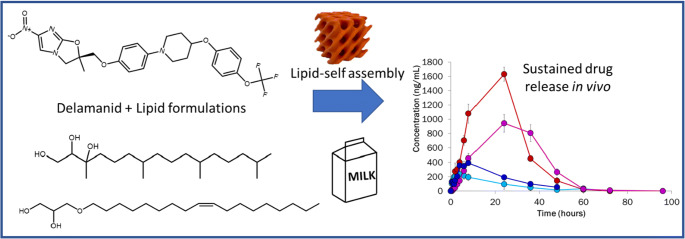
Graphical abstract

## Introduction

**Fig. 1:**
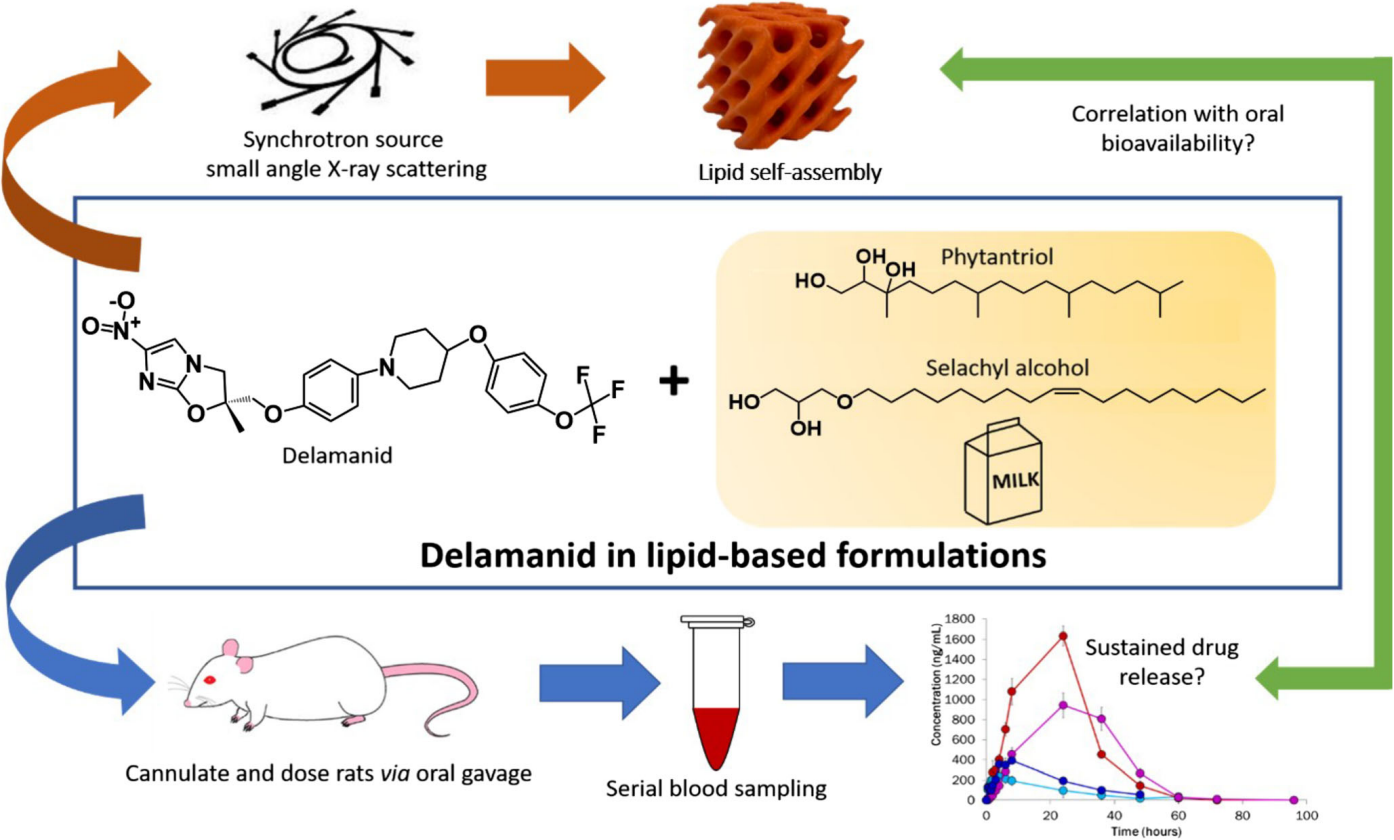
Schematic representation of the concept of this study linking digestibility and lipid self-assembly with pharmacokinetics of delamanid after oral administration

Tuberculosis (TB) caused by *Mycobacterium tuberculosis* remains one of the leading causes of mortality worldwide with over 95% of deaths occurring in low- and middle-income countries [1]. Current first-line treatments for TB typically consist of 6–9 months of oral administration of multiple drugs taken daily or several times a week; and drugs for second-line treatment are administered when resistance to the first-line TB drugs has been diagnosed. Delamanid (previously known as OPC-67683, see Fig. 1 for chemical structure) is a nitro-dihydro-imidazooxazole derivative known to exhibit potency against multidrug-resistant tuberculosis (MDR-TB) strains [2, 3], which can be used in a combination drug treatment for MDR-TB. The recommended dose of commercially available delamanid (Deltyba) in adults is 100 mg daily (two tablets twice a day) for a 6-month period of treatment administered with an appropriate combination drug [4]. Given the complexity of dosing and its likely impact on patient adherence, oral dosage forms for sustained release of drug can potentially be developed to minimise the frequency of dosing and to prevent excessive fluctuations in plasma drug concentrations [5].

Lipids are common excipients that have been used as oral formulations for the delivery of poorly water-soluble drugs [6]. There has been recent interest in lipid systems that can form and maintain ordered liquid crystal structures [inverse bicontinuous cubic phases (V_2_) and inverse hexagonal phases (H_2_)] in the gastrointestinal (GI) environment as a means of extending the duration of drug absorption. While triglyceride-based lipids transiently form these ordered structures in the aqueous environment of the GI tract during digestion [7, 8], other lipids form these structures intrinsically in water and the structures persist on exposure to aqueous gastrointestinal environments. Generally, lipids that are readily and rapidly digestible in the GI tract exhibit rapid drug release while slowly digested or non-digestible lipids that form these structures have been shown to provide a delayed drug release profile [6, 9]. Examples of non-digestible lipids that form ordered phases are phytantriol (PHY) and selachyl alcohol (SA) [10, 11], while glyceryl monooleate (GMO) is a digestible liquid crystal-forming lipid [10, 12]. Phytantriol self-assembles in aqueous environments to form a V_2_ cubic phase while SA forms an H_2_ phase. The non-digestibility of the lipids provides for persistence of the ordered structure in the GI tract, which correlates with gastric retention. The combination of these properties provided an increase in oral bioavailability of the poorly water-soluble drug cinnarizine when administered in PHY or SA, compared with administration in a digestible liquid crystal-forming lipid (GMO) [10, 11].

In this study, we aim to investigate whether prolonged absorption of delamanid could be achieved following administration with the non-digestible lipids PHY and SA, and how it may be linked to the lipid self-assembly behaviour (Fig. 1). The impact of addition of delamanid on the liquid crystalline structures formed by PHY and SA was studied using small angle X-ray scattering (SAXS). The pharmacokinetic behaviour of delamanid released from PHY and SA was subsequently compared with a milk-based formulation as a digestible triglyceride-based comparator.

## Materials and methods

### Materials

Delamanid was purchased from Nanjing Bilatchem Industrial Co. Ltd. (Nanjing, China). Phytantriol (3,7,11,15-tetramethylhexadecane-1,2,3-triol; PHY; 95% purity) was purchased from DSM Nutritional Products (Singapore). Selachyl alcohol (1-O-octadec-9-enyl glycerol; SA; 99% purity) was purchased from Haihang Industry Co., Ltd., (Jinan, China). All lipids were used without further purification. Devondale full cream instant milk powder (trademark of Murray Goulburn Co-operative Co. Ltd.) was purchased from a local supermarket (VIC, Australia). Tween 80, carboxymethylcellulose, Trizma® maleate (reagent grade), 4-bromophenylboronic acid (4-BPBA, > 95% purity), sodium taurodeoxycholate hydrate (NaTDC, > 95% purity) and diazepam were purchased from Sigma-Aldrich (St. Louis, USA). Acetonitrile (HPLC grade), methanol (LiChrosolv) and glacial acetic acid were purchased from Merck Millipore (Bayswater, Australia). Hydrochloric acid (36%) was purchased from Biolab Ltd. (VIC, Australia). Calcium chloride dihydrate (> 99% purity) and sodium hydroxide pellets (min. 97% purity) were purchased from Ajax Finechem (NSW, Australia). Sodium chloride (> 99% purity) was purchased from Chem Supply (SA, Australia). USP grade pancreatin extract was purchased from Southern Biologicals (VIC, Australia). Sodium heparin (1000 IU/mL) was purchased from DBL (VIC, Australia). Saline (0.9% sodium chloride solution) was purchased from Baxter Healthcare Pty. Ltd. (NSW, Australia). Lethabarb (325 mg/mL solution of pentobarbitone sodium) was purchased from Abbott Laboratories Pty. Ltd. (NSW, Australia). DOPC (1,2-dioleoyl-*sn*-glycero-3-phosphatidylcholine) was purchased from Sapphire Bioscience Pty. Ltd. (NSW, Australia). Distilled and deionised water (18.2 MΩ/cm at 25 °C) was obtained from a Millipore water purification system (Billerica, USA).

### Self-assembly behaviour of phytantriol and selachyl alcohol: SAXS studies

Bulk PHY and SA (about 30 mg) were added to 1 mL of water, 0.1 M HCl solution (simulated gastric condition) or bile salt micelle solution (simulated small intestinal condition) with and without delamanid (drug/lipid ratio of 1:10 w/w, selected as a compromise between analytical sensitivity and volume of administration in later in vivo studies), and the samples were incubated at 37 °C for 48 h. The bile salt micelle solution (4.70 mM NaTDC and 0.98 mM DOPC), based on infant fasted conditions [13], was prepared in Tris buffer (50 mM trizma maleate, 5 mM calcium chloride dihydrate, 150 mM sodium chloride and 6 mM sodium azide) at pH 6.5, described in significant detail previously [14]. The self-assembled structures of PHY and SA in the three aqueous solutions were characterised using the SAXS/WAXS beamline at the Australian Synchrotron (ANSTO, VIC, Australia) [15]. Samples were loaded onto a 96-well temperature-controlled plate and sealed using Kapton (polyimide) tape. The plate (held at 37 °C) was mounted in the path of the X-ray beam (photon energy: 12 keV and wavelength: 1.033 Å), and measurements were taken with a 1 s acquisition time. A Pilatus 1 M (170 mm × 170 mm) detector, located approximately 1540 mm from the sample position, was used to generate two-dimensional SAXS patterns, which were reduced to scattering functions plotted as intensity (I) versus scattering vector (*q* = (4π/λ)sinθ where λ = X-ray wavelength and 2θ = scattering angle) using Scatterbrain software version 2.71 developed in-house at the Australian Synchrotron. Suspensions of delamanid in water and the simulated gastric and intestinal solutions containing no PHY or SA were also analysed. Samples were loaded into 1.5 mm outer diameter glass capillaries (Charles Supper, Westborough, MA, USA) and placed on a temperature-controlled capillary holder maintained at 37 °C. SAXS measurements were performed using the parameters described above.

### Solubility of delamanid in lipid-based formulations

The solubility of delamanid in PHY, SA and milk (9% fat to match the dose of PHY/SA, prepared by reconstituting milk powder in Tris buffer) was measured to estimate drug loading for the in vivo pharmacokinetic studies. To determine the equilibrium solubility of delamanid in PHY and SA, excess delamanid was added into microcentrifuge tubes containing molten PHY and SA (prepared by incubating the lipids at 37 °C). The lipid + drug mixtures were vortexed and incubated at 37 °C on a roller mixer. Samples were removed after 4, 7 and 10 days of incubation, and centrifuged at 16,162*g* for 30 min. Approximately 100 mg of the upper lipid layer was transferred into ultracentrifuge tubes, and the samples were re-centrifuged at 434,902*g* for 60 min at 37 °C. About 20 mg of the lipid supernatant layer was collected and dissolved in 1:1 tetrahydrofuran:acetonitrile volume ratio. The samples were further diluted with acetonitrile (1:1 volume ratio) prior to injection into a Shimadzu (Shimadzu, Kyoto, Japan) HPLC system consisting of a CBM-20A system controller, an LC-30AD solvent delivery module, a SIL-30AC auto-sampler and a CTO-20AC column oven coupled with an SPD-M30A diode array UV detector set to integrate at 254 nm. Chromatographic separation of delamanid was performed at 35 °C on a Waters Symmetry C_18_ column (3.5 μm, 100 Å, 4.6 × 75 mm). Delamanid was assayed using a binary gradient elution with a flow rate of 0.5 mL/min: 30–90% B for 10 min, 90–30% B for 3 min and 30% B for 4 min. Buffer A was 1% v/v glacial acetic acid in water, and buffer B was 1% v/v glacial acetic acid in acetonitrile. Delamanid stock solutions (in ACN) were used to provide a standard reference range of 0.1–40 μg/mL in ACN. The injection volume was 10 μL and the retention time for delamanid was 6.9 min.

To determine the equilibrium solubility of delamanid in high fat (9% *w*/*v*) reconstituted milk, excess delamanid was added to the milk. The drug + milk mixtures were vortexed and incubated at 4 °C with constant stirring. Samples (200 μL) were removed at specified time points (3 h, 24 h and 3 days) and were placed into ultracentrifuge tubes and ultracentrifuged at 434,902*g* for 60 min at 25 °C. The resultant aqueous, lipid and pellet layers were collected separately followed by extraction of delamanid using acetonitrile (for the aqueous layer) or 1:4 v/v methanol/acetonitrile (for the lipid and pellet layers) containing diazepam as the internal standard for HPLC. The amount of delamanid partitioned into the individual digested phases was quantified using the HPLC method described in the “Quantification of delamanid in plasma using HPLC” section.

### Bioavailability of delamanid in rats

#### Preparation of formulations for oral administration

Delamanid was loaded into bulk PHY, bulk SA and milk, at a drug/lipid ratio of 1:10 w/w and a fixed dose of 10 mg delamanid per kilogram of rat. Aqueous suspensions of delamanid (containing 0.5% v/v sodium carboxymethylcellulose and 0.4% v/v Tween 80 in saline) were also prepared as control formulations. The drug aqueous suspensions and the lipid-containing formulations were equilibrated for 24 h at 37 °C prior to dosing.

#### Animal procedures and sample collection

All animal studies were approved and conducted in accordance with the guidelines of the Monash Institute of Pharmaceutical Sciences Animal Ethics Committee. Male Sprague Dawley rats (280–320 g) were used for the pharmacokinetic studies, and the rats were divided into 4 treatment groups with 4 rats per treatment: delamanid in aqueous suspension, PHY, SA and milk formulations. Rats were anaesthetised via inhalation of 5% isoflurane (and maintained at 2% for the duration of the procedure). The right carotid artery was isolated and cannulated with a 0.80 mm OD × 0.50 mm ID polyethylene tubing to allow serial blood sampling. Cannulas were filled and flushed with 10 IU/mL heparin in normal saline to maintain cannula patency. The cannula was tunnelled to back of the neck. The rats were then placed in a tether swivel system in individual wire bottom metabolic cages to recover overnight prior to dosing and blood sampling. The rats were then fasted for at least 12 h prior to dosing and 8 h after dosing, with water provided ad libitum. The lipid formulations and the saline suspensions were dosed via oral gavage (225 μL), with the dose accurately calculated by weighing the gavage and syringe before and after dosing. Serial blood sampling was performed at 0.5, 1, 1.5, 2, 3, 4, 6, 8, 24, 36, 48, 60, 72 and 96 h (up to 48 h for milk) with 250 μL drawn at each time point. Blood samples were dispensed in 1.5 mL microcentrifuge tubes containing 10 IU sodium heparin, which were then centrifuged for 5 min at 6700*g*. Aliquots of plasma (50 μL) were collected in duplicates, and samples were kept at − 20 °C until quantification of delamanid using HPLC.

#### Quantification of delamanid in plasma using HPLC

The collected plasma samples (50 μL) were spiked with 10 μL of diazepam solution (10 μg/mL in acetonitrile) as an internal standard followed by the addition of 90 μL acetonitrile. Samples were then centrifuged at 16,162*g* for 7 min at room temperature, and 95 μL of the supernatant were transferred to HPLC vials. Plasma standards were prepared by spiking delamanid stock solutions (in ACN) into plasma to give a standard reference range of 5–1000 ng/mL. The injection volume was 50 μL. Chromatographic separation of delamanid was performed using HPLC methods described in the “Solubility of delamanid in lipid-based formulations” section with slight modification to the elution gradient: 30–50% B for 7 min, 50% B for 0.5 min, 50–70% B for 2.5 min, 70% B for 1 min, 70–90% B for 1 min, 90–30% B for 1 min and 30% B for 4 min. The retention time for diazepam was 9.6 min and for delamanid was 10.3 min.

#### Determination of pharmacokinetic parameters

Concentrations of delamanid in plasma were dose-normalised to 10 mg/kg to account for variations in animal weight. Values of C_max_ (maximum drug concentration in plasma) and T_max_ (time to reach C_max_) were determined from the normalised data. The truncated area under the curve (AUC) from 0 to the last measurable time points (AUC_0-tlast_) was determined using the trapezoidal rule. SPSS for Windows (Version 23) was used to statistically determine differences in the data using one-way ANOVA analysis of variance with Tukey’s multiple comparison, assuming statistical significance when *p* value was ≤ 0.05.

### Solubilisation of delamanid in milk during in vitro lipolysis

In vitro lipolysis of milk mixed with delamanid was performed to use the in vitro solubilisation data to interpret the bioavailability of delamanid. Delamanid (22.5 mg) was added to 2.5 mL of water containing 0.25 mL of 1 M HCl solution. The drug sample was vortexed thoroughly prior to the addition of 17.5 mL of reconstituted powdered milk (9% fat in the Tris buffer) containing bile salt micelles with final concentrations of 4.7 mM NaTDC and 0.98 mM DOPC. The drug + milk mixture (22.5 mg delamanid/1.58 g milk fat) was incubated in a thermostated (37 °C) glass vessel under constant magnetic stirring. The pH of the mixture was adjusted to 6.500 ± 0.005 prior to the initiation of lipolysis with pancreatin lipase suspension (2.25 mL, ~700 TBU/mL of digest). The pancreatin lipase solution was prepared from pancreatin extract using methods previously described [16]. The pH of the sample in the digestion vessel was maintained at 6.5 during digestion using 2 M NaOH solution titrated into the digesta by a pH STAT control module (Metrohm AG, Herisau, Switzerland). Samples (200 μL) were collected before (0 min) and after digestion (60 min) into ultracentrifuge tubes, and 2 μL of lipase inhibitor (0.5 M 4-BPBA in methanol) was added into each sample before ultracentrifugation at 434,902*g* for 1 h at 37 °C. The resultant aqueous, lipid and pellet layers were collected separately after ultracentrifugation and extraction of delamanid from the three separate layers were performed using methods described in the “Solubility of delamanid in lipid-based formulations” section. Delamanid concentrations in each separated layer were determined based on the standard curve made by spiking delamanid stock solutions (in ACN) in digested milk with concentrations ranging from 0.1–250 μg/mL. The delamanid concentration was quantified using the HPLC method described in the “Quantification of delamanid in plasma using HPLC” section.

## Results and discussion

### Self-assembly behaviour of phytantriol and selachyl alcohol in the presence of delamanid

**Fig. 2:**
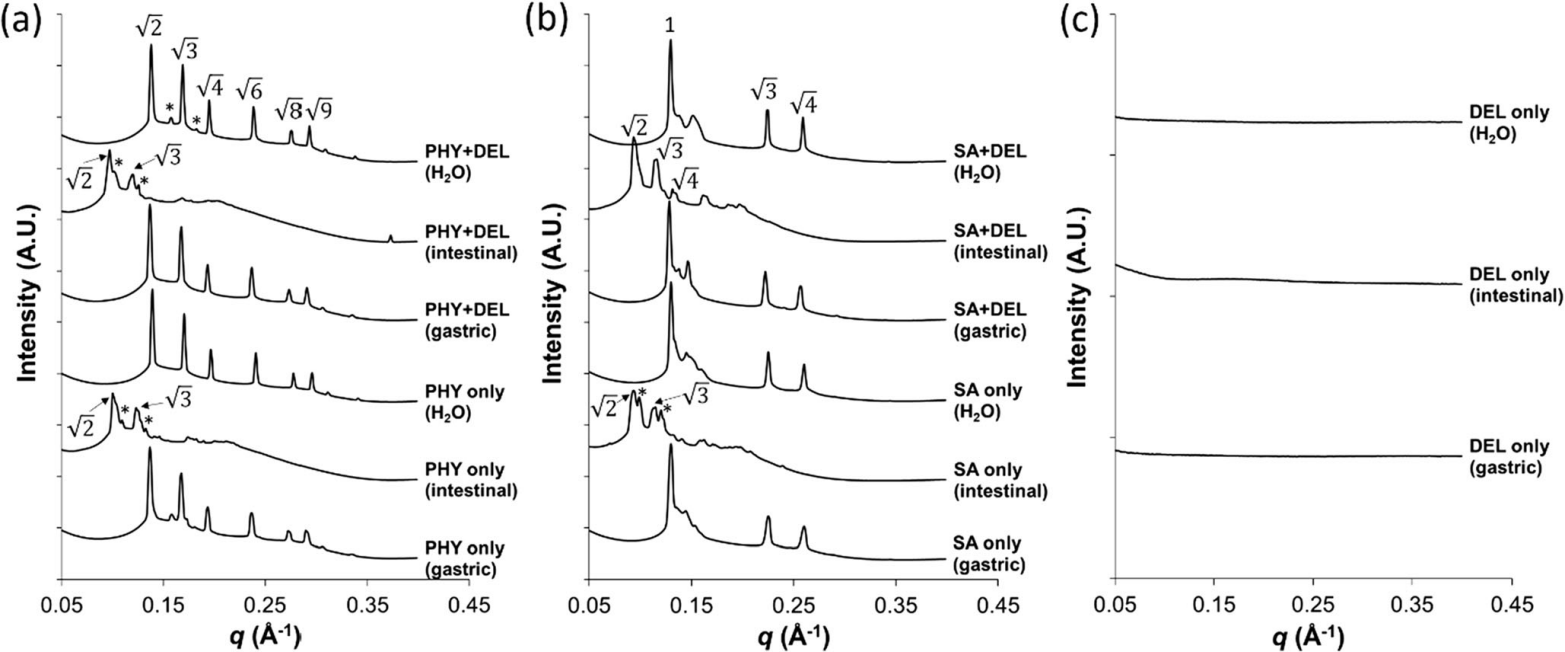
Effect of aqueous environment and the presence of delamanid on the self-assembly of the lipid delivery vehicles measured using X-ray scattering. Panel **a** bulk phytantriol (PHY) and **b** selachyl alcohol (SA) in excess water, simulated gastric and small intestinal conditions in the absence and presence of delamanid (DEL). Panel **c** shows the X-ray scattering profiles for delamanid suspensions over the same range of scattering vector *q*. The ratio of the Bragg peaks for the V_2_ phase with *Pn*3*m* space group, and the H_2_ phase were identified. For *Pn*3*m*, the peak ratios were $\sqrt{2}$, $\sqrt{3}$, $\sqrt{4}$, $\sqrt{6}$, $\sqrt{8},\sqrt{9}$; and for H_2_ phase, the peak ratios were 1, $\sqrt{3}$ and $\sqrt{4\ }$ [17, 18]. Peaks labelled with “*” were indicative of a *Pn*3*m* structures with different lattice parameters

Formation of lipid liquid crystal structures in the gastrointestinal tract is known to dictate the partitioning of poorly water-soluble drugs and their oral bioavailability [9]. Characterising the self-assembly properties of PHY and SA in the gastrointestinal condition and the impact of incorporated drug is therefore important in understanding how these lipids behave during their passage through the gastrointestinal tract. The phase behaviour of bulk PHY and SA after exposure to different aqueous solution conditions (water, simulated gastric and simulated small intestinal conditions) was characterised using synchrotron SAXS to confirm the formation of lyotropic liquid crystalline structures due to self-assembly of the amphiphilic molecules. The scattering profiles in Fig. 2 show that an inverse bicontinuous cubic phase (V_2_) with a *Pn*3*m* space group was formed after hydration of PHY in excess water as observed in previous studies [11, 19]. In comparison, SA self-assembled in water to form an inverse hexagonal phase [20] (H_2_; relative peak ratios of 1, $\sqrt{3}$ and $\sqrt{4}$) [17], although H_2_ phases with smaller lattice parameters relating to non-equilibrium structures were also present (see Table 1). These liquid crystal structures were not significantly impacted by the addition of delamanid. Similarly, no structural changes occurred following incubation of PHY and SA with and without delamanid at low pH (gastric) conditions, which was not unexpected considering the non-ionic nature of the lipids.

**Table 1:** Predominant liquid crystal structures and their corresponding lattice parameters for the self-assembly of phytantriol (PHY) and selachyl alcohol (SA) in excess water with and without delamanid at low pH (gastric) and intestinal pH containing bile salt micelles

System	Condition	Predominant structure	Lattice parameter (Å)
PHY	Water	*Pn*3*m*	64
	Gastric	*Pn*3*m*	65
	Intestine	*Pn*3*m*	87
PHY + delamanid	Water	*Pn*3*m*	64
	Gastric	*Pn*3*m*	65
	Intestine	*Pn*3*m*	92
SA	Water	H_2_	55
	Gastric	H_2_	56
	Intestine	*Pn*3*m*	95
SA + delamanid	Water	H_2_	56
	Gastric	H_2_	56
	Intestine	*Pn*3*m*	94

Lattice parameters for the minor peaks in the SAXS profiles due to the presence of V_2_ phases with the *Ia*3*d* space group are described in Table S1

Figure 2 shows the influence of bile salt micelles (NaTDC/DOPC) on the lyotropic liquid crystal structures formed by self-assembly of PHY and SA. Changes in structure of the V_2_ and H_2_ phases occurred following addition of surfactants at an intestinal pH of 6.5. For PHY, the *Pn*3*m* phase remained stable but there was an increase in lattice parameter of the *Pn*3*m* phase from 64 to 87 Å (92 Å for PHY with delamanid). For SA, the H_2_ structure transformed into to a *Pn*3*m* cubic phase upon addition of bile salt micelles (Table 1). Addition of bile salts to PHY and SA therefore resulted in a less negative lipid curvature that could arise due to interaction between the bile acids and the lipid components, increasing the average size of the polar headgroups, and/or by decreasing the rigidity of the lipid bilayers [21]. It should be noted that the amount of bile salt micelles added to the aqueous buffer was within the concentration range generally reported for simulated human fasted intestinal fluid [22] and may not reflect the in vivo conditions in rats, in which higher concentrations of bile acids up to about 50 mM have been observed [23]. The high concentrations of bile salts could therefore potentially further reduce the negative curvature of the lipids from *Pn*3*m* and H_2_ towards complete incorporation of PHY and SA into the bile salt mixed micelles, completely disrupting the inverse liquid crystalline phases.

### Pharmacokinetic studies of delamanid

**Fig. 3:**
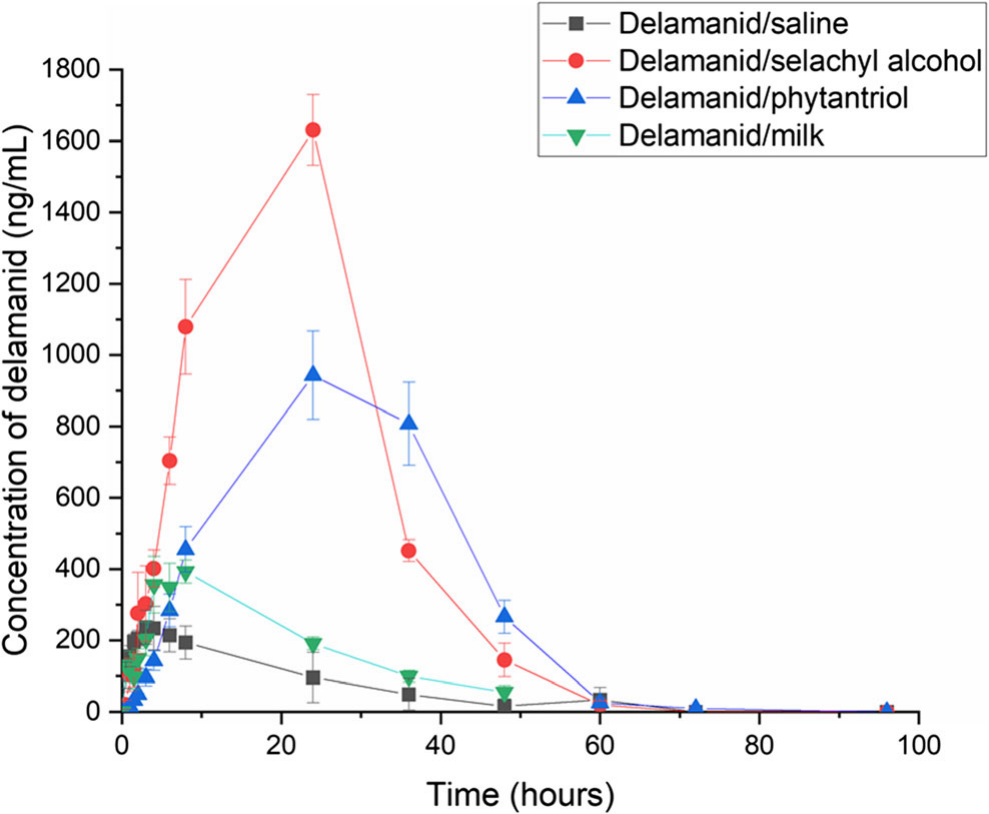
Mean dose-normalised plasma concentrations of delamanid following oral administration in aqueous saline suspension and lipid-based formulations (phytantriol, selachyl alcohol and milk) over 96 h. Data are presented as mean ± SEM (*n* = 4)

Mean concentrations of delamanid in plasma after oral administration in saline solution, PHY, SA and milk are shown in Fig. 3. Prolonged and increased levels of exposure to delamanid could be observed for all the lipid-based formulations compared with the saline suspension, particularly when administered with SA and PHY. The extent of drug exposure (determined by the area under the curve in the plasma concentration-time plot) and the maximum concentration of delamanid (C_max_) achievable at a specific time point (T_max_) are summarised in Table 2.

**Table 2:** Pharmacokinetic parameters (mean ± SEM, *n* = 4) after oral administration of delamanid in aqueous suspension and delamanid in lipid formulations dosed at 10 mg delamanid per kilogram of rat

Formulation	T_max_ (hr)	C_max_ (ng/mL)	AUC_0-last_ (hr ng/mL)	Relative bioavailability (to suspension)
Aqueous saline suspension	3.4 ± 1.0	256 ± 45	5680 ± 2530	100
Phytantriol	27.0 ± 3.0^**^	1055 ± 29^***^	31,600 ± 2230^*^	556
Selachyl alcohol	20.0 ± 4.0^**^	1586 ± 93^****^	36,600 ± 5080^**^	645
Milk	6.5 ± 1.0	412 ± 38	9170 ± 613	162

*p < 0.05 when compared with the saline suspension

**p < 0.05 when compared with the saline suspension and milk

***p < 0.05 when compared with the saline suspension and selachyl alcohol

****p < 0.05 when compared with the saline suspension, phytantriol and milk

C_max_ maximum drug concentration in plasma; T_max_ time to reach C_max_; and AUC_0-last sample_ area under curve of plasma drug concentration vs time truncated from time 0 to the last time point sampled at 48 h for milk and 96 h for the other formulations

Overall, the relative bioavailability of the lipid formulations, calculated by the ratio of the AUC in the lipid formulations to delamanid aqueous saline suspension after dose normalisation, was highest for SA followed by PHY and milk. Analysis of the pharmacokinetic parameters using ANOVA revealed statistical differences in the C_max_, T_max_ and AUC_0-last_ between PHY, SA, and delamanid suspensions with *p* values ≤ 0.05. However, no statistical differences were observed in the T_max_ and AUC_0-last_ (*p* values > 0.05) between delamanid in the aqueous suspension and milk, although the mean C_max_ for delamanid in milk was significantly greater.

As shown in Table 2, the mean T_max_ was shortest when delamanid was administered in aqueous saline and milk suspensions. This indicated a rapid absorption of delamanid from the saline and milk suspensions compared with the other two formulations, although the lower mean C_max_ and AUC_0-last_ meant that delamanid was less bioavailable from the milk and aqueous suspension when compared with PHY and SA.

The solubility of delamanid in PHY and SA was experimentally determined to be ~ 0.14 ± 0.01 and 0.41 ± 0.03 mg drug/g lipid, respectively, compared with 3.29 ± 0.04 mg/g milk lipid (overall solubility in reconstituted milk at 9% fat was ~ 0.38 ± 0.01 mg/mL). The lower drug exposure from the milk formulation may therefore be attributed to the loss of drug solubilisation capacity following digestion of milk due to the formation of increasingly hydrophilic lipids during digestion. Digestion of the lipids in milk and the release of fatty acids, which have been previously shown to improve bioavailability of other poorly water-soluble drugs [24], were apparently insufficient to support the complete solubilisation of delamanid, despite the higher solubility of DEL in undigested milk compared with PHY and SA.

To further support the findings that digestion of milk did not result in improved drug solubilisation, in vitro digestion of delamanid in the reconstituted powdered milk (9% fat w/v) was performed and the amount of drug partitioned into the lipid and aqueous phases of the milk was quantified. Results in Fig. S1 showed that the amount of delamanid partitioned into the lipid and aqueous phases of milk before and after 60 min of digestion were not significantly different (*p* value > 0.05). The solubilisation capacity of delamanid in the digested milk at the 60 min time-point was also not statistically significantly different to the equilibrium solubility of delamanid in 9% fat milk. It was therefore likely that digestion of milk did not improve the solubility of delamanid; hence, there was no substantial improvement in oral bioavailability using the milk formulation.

The pharmacokinetic profiles for PHY and SA showed that absorption of delamanid occurred for an extended period of time, and a significant increase in the oral bioavailability was realised. Delamanid was formulated in PHY and SA bulk lipid at a 10:1 lipid:drug mixture, which was in excess of the measured equilibrium solubilities. This meant that excess drug crystals were present due to low drug solubility. It has been previously demonstrated that PHY and SA are retained in the stomach for extended periods of time (> 24 h for PHY) [10]. Previous studies with these lipids have typically had drug in solution in the lipid which resulted in T_max_ values of 33.0 ± 5.0 and 23.5 ± 5.9 h, respectively [10]. However, one study incorporated gold nanoparticles in PHY that were also retained in the stomach for extended periods (> 8 h) compared with a digestible lipid system when measured by X-ray computer tomographic imaging [25]. Thus it is not unexpected that a suspension of drug in these lipids is also likely to be retained for an extended period of time in the stomach. The chemical stability of PHY and SA against digestive enzymes appears to prevent these systems from exiting the stomach as quickly as digestible lipid systems or aqueous suspensions. The gastric compartment then has the potential to act in a non-sink condition, requiring drug released from the lipid suspension matrix to leave the stomach before further dissolution and partitioning can occur into the gastrointestinal fluids. Thus, the long duration of release can be understood through likely gastric retention already well demonstrated for these self-assembling lipid systems, but for the first time here with drug in suspension rather than fully dissolved.

Perhaps of greater interest are the differences in C_max_ and AUC for the SA formulation compared with the PHY formulation. There is more drug in solution in the SA formulation than the PHY formulation by virtue of the greater solubility of delamanid in the host lipid, so it is likely that some lipid erosion occurs together with the drug release described above, leading to more drug arriving in the intestine in a dissolved state and commensurately greater absorption. The kinetic profiles are very similar, indicating that there are likely differences in the amount of drug in solution and available for absorption and that this drives the differences in plasma concentrations of drug over time.

While the formation of self-assembled structures using non-digestible lipids has been previously shown to be critical in observing the long duration gastric retention behaviour, the type of liquid crystal structure formed does not appear to play a major role in the process. The self-assembly of the lipids under intestinal conditions was the same (both formed the *Pn3m* cubic phase under intestinal conditions, Table 1) so phase behaviour in the intestine is unlikely to have played a major role in the differences in overall exposure observed between the PHY and SA formulations. The similar shape of the kinetic profiles also suggests that the differences in phase behaviour in the gastric conditions (inverse hexagonal phase vs cubic phase) did not appear to play a significant role either. Thus, it is the propensity to form a liquid crystalline structure with non-digestible lipids, rather than the lipid and structure per se that appears to be important.

The PHY and SA formulations show a clear ability to provide enhanced drug exposure over at least a 24 h period. In the context of reducing pill burden and the potential for co-formulation with a second once-daily drug in combination with delamanid, the lipid formulations studied here have potential to enable improved administration regimes including once daily dosing, which would provide an important opportunity to simplify treatment for tuberculosis in challenging populations. The two non-digestible lipids investigated here do not yet have GRAS status, although phytantriol [26] is a commercial cosmetic ingredient and selachyl alcohol [27] is an endogenous lipid also used in cosmetics. The oral LD50 values of phytantriol and related compounds are high > 2000 mg/kg in rats, so it is unlikely that they would show unusual toxicity behaviour; however, this remains to be determined in future formal preclinical and clinical safety studies.

## Conclusions

This study showed the ability of phytantriol (PHY) and selachyl alcohol (SA) to sustain the release of delamanid from lipid formulations when administered in rats. The simple approach incorporates the non-digestible nature of lipids with prolonged gastric retention. In contrast, oral administration of delamanid with digestible lipids in the form of milk did not show sustained drug release characteristics. Comparable drug exposure between milk and an aqueous saline formulation was seen, which also highlighted the limited effect of milk fat digestion on the absorption of delamanid. Our findings suggested the potential use of non-digestible lipids as formulation strategies to improve the oral bioavailability of delamanid and extend the duration of drug exposure in plasma, with the potential to improve patient compliance by reducing the frequency of treatment.

## Electronic supplementary material

ESM 1 (DOCX 97.4 kb)
