# Supplementary material for: Sustained absorption of delamanid from lipid-based formulations as a path to reduced frequency of administration
Source: Drug Deliv Transl Res. 2020 Sep 15;11(3):1236–44. doi: 10.1007/s13346-020-00851-z (PMC8096769; doi:10.1007/s13346-020-00851-z)
Supplement: Supplementary file 1 — (DOCX 97.4 kb) [file 13346_2020_851_MOESM1_ESM.docx]

**Supporting Information**

**Sustained absorption of delamanid from lipid-based formulations as a path to reduced frequency of administration**

Gisela Ramirez,^1^ Anna Pham,^1^ Andrew J. Clulow,^1^ Malinda Salim,^1^ Adrian Hawley,^2^ Ben J. Boyd*^1,3^

^1^Drug Delivery, Disposition and Dynamics, Monash Institute of Pharmaceutical Sciences, Monash University (Parkville Campus), 381 Royal Parade, Parkville, VIC 3052, Australia

^2^SAXS/WAXS beamline, Australian Synchrotron, ANSTO, 800 Blackburn Rd, Clayton, VIC 3169, Australia

^5^ARC Centre of Excellence in Convergent Bio-Nano Science and Technology, Monash Institute of Pharmaceutical Sciences, Monash University (Parkville Campus), 381 Royal Parade, Parkville, VIC 3052, Australia

*Corresponding author details:

Postal address: Monash Institute of Pharmaceutical Sciences, Monash University (Parkville Campus), 381 Royal Parade, Parkville, VIC 3052, Australia

Telephone: +61 3 99039112; Fax: +61 3 99039583

Email: [ben.boyd@monash.edu](mailto:ben.boyd@monash.edu)

**

**

**Figure S1**. Amount of delamanid present in the supernatant phase of tris buffer, and supernatant + lipid phases for 9% milk before (0 min) and after (60 min) digestion. Average equilibrium solubility of delamanid in 9% milk was shown by the horizontal dashed line.

**Table S1.** Liquid crystal structures and their corresponding lattice parameters for the self-assembly of phytantriol (PHY) and selachyl alcohol (SA) in excess water with and without delamanid at low pH (gastric) and intestinal pH containing bile salt micelles.

| System | Condition | Structure | Lattice parameter (Å) |
| --- | --- | --- | --- |
| PHY | Water | *Pn*3*m* | 64 |
|  | Gastric | *Pn*3*m* + *Ia*3*d* (traces) | 65 (*Pn*3*m*) 97 (*Ia*3*d*) |
|  | Intestine | *Pn*3*m* | 87 (and traces at 82) |
| PHY + delamanid | Water | *Pn*3*m* + *Ia*3*d* (traces) | 64 (*Pn*3*m*) 97 (*Ia*3*d*) |
|  | Gastric | *Pn*3*m* | 65 |
|  | Intestine | *Pn*3*m* | 92 (and traces at 88) |
| SA | Water | H_2_ | 55 (and traces at 50) |
|  | Gastric | H_2_ | 56 (and traces at 50 and 47) |
|  | Intestine | *Pn*3*m* | 95 (and traces at 90) |
| SA + delamanid | Water | H_2_ | 56 (and traces at 53 and 48) |
|  | Gastric | H_2_ | 56 (and traces at 53 and 49) |
|  | Intestine | *Pn*3*m* | 94 |
